# Escape from IFN-γ-dependent immunosurveillance in tumorigenesis

**DOI:** 10.1186/s12929-017-0317-0

**Published:** 2017-02-01

**Authors:** Chiou-Feng Lin, Chih-Ming Lin, Kang-Yun Lee, Szu-Yuan Wu, Po-Hao Feng, Kuan-Yuan Chen, Hsiao-Chi Chuang, Chia-Ling Chen, Yu-Chih Wang, Po-Chun Tseng, Tsung-Ting Tsai

**Affiliations:** 1 Department of Microbiology and Immunology, School of Medicine, College of Medicine, Taipei Medical University, Taipei, 110 Taiwan; 2 Graduate Institute of Medical Sciences, College of Medicine, Taipei Medical University, Taipei, 110 Taiwan; 3 Department of Thoracic Surgery, Lotung Poh-Ai Hospital, Yilan, 265 Taiwan; 4 Division of Pulmonary Medicine, Department of Internal Medicine, Shuang Ho Hospital, Taipei Medical University, Taipei, 110 Taiwan; 5 Department of Internal Medicine, School of Medicine, College of Medicine, Taipei Medical University, Taipei, 110 Taiwan; 6 Graduate Institute of Toxicology, College of Medicine, National Taiwan University, Taipei, 100 Taiwan; 7 Department of Radiation Oncology, Wan Fang Hospital, Taipei Medical University, Taipei Medical University, Taipei, 110 Taiwan; 8 Department of Biotechnology, Hung Kuang University, Taichung, 433 Taiwan; 9 Graduate Institute of Clinical Medicine, College of Medicine, Taipei Medical University, Taipei, 110 Taiwan; 10 School of Respiratory Therapy, College of Medicine, Taipei Medical University, Taipei, 110 Taiwan; 11 Translational Research Center, Taipei Medical University, Taipei, 110 Taiwan

**Keywords:** Cancer, Immunosurveillance, IFN-γ, Hyporesponsiveness, Escape

## Abstract

Immune interferon (IFN), also known as IFN-γ, promotes not only immunomodulation but also antimicrobial and anticancer activity. After IFN-γ binds to the complex of IFN-γ receptor (IFNGR) 1-IFNGR2 and subsequently activates its downstream signaling pathways, IFN-γ immediately causes transcriptional stimulation of a variety of genes that are principally involved in its biological activities. Regarding IFN-γ-dependent immunosurveillance, IFN-γ can directly suppress tumorigenesis and infection and/or can modulate the immunological status in both cancer cells and infected cells. Regarding the anticancer effects of IFN-γ, cancer cells develop strategies to escape from IFN-γ-dependent cancer immunosurveillance. Immune evasion, including the recruitment of immunosuppressive cells, secretion of immunosuppressive factors, and suppression of cytotoxic T lymphocyte responses, is speculated to be elicited by the oncogenic microenvironment. All of these events effectively downregulate IFN-γ-expressing cells and IFN-γ production. In addition to these extrinsic pathways, cancer cells may develop cellular tolerance that manifests as hyporesponsiveness to IFN-γ stimulation. This review discusses the potential escape mechanisms from IFN-γ-dependent immunosurveillance in tumorigenesis.

## Background

Tumorigenesis is a complicated pathogenesis characterized by the hallmarks of cancer development such as [1] causing instability and mutation in genome, [2] resisting cell death response, [3] deregulating the requirement of cellular energetics, [4] sustaining proliferative signaling pathways, [5] evading growth suppressor response, [6] avoiding immune recognition and destruction, [7] enabling replicative immortality ability, [8] facilitating tumor-promoting microenvironment, [9] activating invasion and metastasis processes, and [10] inducing angiogenic status. Regarding malignancies that are derived from immortalized and transformed cells whose genomes have become altered or mutated, a variety of oncogenic alterations (including activation of the PI3K/AKT and Ras/Raf/MEK/ERK pathways as well as growth factor receptors) and either the inactivation or decreased expression of tumor suppressors such as p53, adenomatous polyposis coli mutations, phosphatase and tensin homolog deleted on chromosome 10 (PTEN), and runt-related transcription factor 3 are pathologically contributed to tumor growth and survival [33]. For immune-based anticancer defense, the immunosurveillance processes of elimination act as tumor-killing defenders; however, through immunoediting, cancer cells initially trigger tolerance, senescence, and/or equilibrium with the immune system followed by the induction of immune escape to promote malignant carcinogenesis [21, 22]. The early stages of most cancers are asymptomatic; however, cancer cells are proliferated and then invaded into the lymph nodes and nearby organs leading to mortality. For immune evasion, tumors develop a number of escape strategies from host surveillance [3, 50]. Despite cooperating with tumor-infiltrating immune cells harboring the immunosuppressive activities such as tumor-associated macrophages (TAMs), cluster of differentiation (CD) 4^+^ CD25^+^ forkhead box P3 (FOXP3)^+^ regulatory T cells (Treg), and myeloid-derived suppressor cells (MDSCs) [7], it is unclear whether oncogenic processes are also involved in the immune escape of cancer cells by inducing a cellular immune tolerance in response to immune recognition and activation.

### Cancer immunosurveillance

Through the immune system, immunosurveillance confers specific and immediate recognition not only of pathogen-infected cells but also of healthy cells that have been immortalized and/or transformed during tumorigenesis [44]. As is known, a variety of cancerous events, such as chemical and infectious carcinogens, hazardous radicals, and carcinogenetic alterations, cause tumorigenesis by altering the expression and/or activation of oncogenes as well as the inactivation of tumor suppressor genes [33]. Under immunosurveillance, immune cells such as T cells, natural killer (NK) cells, NKT cells, γδ T cells, and macrophages functionally translocate into tumor sites and trigger anticancer immunity by secreting several cytotoxic molecules including interferon (IFN)-γ, tumor necrosis factor (TNF)-α, perforin, granzyme, CD95 ligand (FasL), and TNF-related apoptosis-inducing ligand (TRAIL) [21, 22, 69]. An increased cytotoxic T lymphocyte (CTL) response confers better survival against cancers, while the suppression of CTLs increases the susceptibility of the host to carcinogenesis, indicating a major anticancer immunity due to CTLs [31]. Concurrently, infiltrated NK, NKT, and γδ T cells also display anticancer immunity [24, 32, 44, 69].

After cell-cell contact recognition, most anticancer-related cytotoxic and immune modulatory factors secreted from CTLs, NK/NKT cells, and γδ T cells can target cancer cells to directly induce cancer cell apoptosis and/or to sensitize the cancer cell response to apoptotic stimuli. Through a perforin-delivered intracellular granzyme B system, cancer cells can be disrupted by granzyme B-mediated cellular toxicity via different pathways, which subsequently results in cancer cell damage toward immunological cell death [51, 68]. In addition to cytolytic perforin and granzyme B, death ligands such as the CD95 ligand and TRAIL, which are mainly secreted from activated CTLs and NK cells, also influence anticancer immunity [41, 51, 72]. Upon activation of CLTs and NK cells, the CD95 ligand and TRAIL are de novo synthesized and either expressed on the surface of CTLs and NK cells or released via an exosome-mediated pathway to kill susceptible tumor cells through the interaction of these ligands with their respective death receptors. Cancer immunosurveillance allows for the elimination of immortalized and transformed cells from cancerous regions.

### IFN-γ-dependent cancer immunosurveillance

Either type I IFN-α/-β or type II immune IFN-γ are potent cytokines that are cytoprotective against tumorigenesis [57]. Similar to perforin-deficient mice, IFN-γ-deficient mice spontaneously develop lung epithelial malignancies and lymphoma [66], confirming the anticancer ability of IFN-γ. Similarly, IFN-γ receptors (IFNGRs)- and signal transducer and activator of transcription (STAT) 1-deficient mice advance tumor growth after chemical carcinogen treatment. Furthermore, exogenous administration of IFN-γ is used for treating patients with ovarian cancer, adult T cell leukemia and malignant melanoma [53]. Decreased levels of IFN-γ and/or the generation of genetic defects in IFN-γ signaling factors, including single nucleotide polymorphisms in IFN-γ, IFN-regulating factors (IRFs), and its receptor IFNGR2, are risk factors for tumorigenesis in humans [70]. In addition to cytotoxic factors, IFN-γ secreted from CTLs, NK cells, NKT cells, and γδ T cells acts as a potent anticancer cytokine [54, 69]. IFN-γ exhibits a variety of important biological activities: not only does IFN-γ confer antimicrobial and immunomodulatory effects—inducing MHC-mediated antigen presentation pathways, developing type 1 T helper cell (Th1) responses, causing anti-microbe effects, regulating leukocyte trafficking, and facilitating Toll-like receptor signaling—but it also promotes anticancer activities [65]. IFN-γ binds IFNGR1 and IFNGR2, which are associated with Janus kinase (JAK) 1 and JAK2, respectively. Activated JAKs cause tyrosine phosphorylation of STAT1 followed by the formation of STAT1-STAT1 homodimers. In addition to JAKs, IFN-γ causes p38 mitogen-activated protein kinase (MAPK) activation to mediate the phosphorylation of Ser727 on both STAT1 and STAT3 [46]. After activation, STATs translocate into the nucleus and bind to IFN-γ-activated site (GAS) elements for initiating the transcription of several genes related to anticancer such as major histocompatibility complex (MHC) class I, CD95 (Fas), caspase-1 and other genes associated with growth inhibition [60].

IFN-γ displays anticancer activity by attenuating cancer cell growth. After IFN-γ stimulation, p21 and p27 are expressed to arrest the cell cycle by attenuating the stability of cyclin/cyclin-dependent kinase complexes [34, 45]. In addition, IFN-γ increases the expression of miRNAs to contribute to p53-regulated cell cycle arrest [49, 63]. Through a direct effect, IFN-γ induces cell apoptosis via Bcl-2 downregulation [84]. The activation of cathepsin, generation of reactive oxygen species (ROS), and induction of endoplasmic reticulum (ER) stress are involved in the apoptotic signaling of IFN-γ [86]. Exogenous administration of IFN-γ causes a mimic extracellular trap cell death (ETosis) in A549 adenocarcinoma cells [47, 48]. In IFN-γ-treated lung epithelial malignancies, IFN-γ induces autophagy in IFN-inducible immunity-related p47 GTPase IRGM1- and activating transcription factor 6-regulated manners. The induced autophagosome may serve as a platform for Atg5/Fas-associated protein with death domain-mediated caspase-8/caspase-3 activation, while IFN-γ induces IRF-1-mediated caspase cascade activation. Caspase-mediated lamin A/C degradation causes DNA damage followed by ataxia telangiectasia mutated (ATM) and ATR (ATM and Rad3-related) activation and γ-H2AX phosphorylation. Through an unknown mechanism, ATR/ATM regulates protein arginine deiminase (PAD) 4-mediated histone H3 citrullination and ETosis. In addition, NADPH oxidase/ROS signaling induced by IFN-γ also facilitates DNA damage and ETosis. IFN-γ-induced ER stress causes the accumulation of intracellular calcium, which contributes to PAD4 activation and ETosis. However, the potential role of IFN-γ-induced mimetic ETosis in cancer cells remains unclear.

In addition to the direct cytotoxic and growth inhibitory effects of IFN-γ, IFN-γ may facilitate anticancer immunity through its immunomodulatory actions. In patients with lung cancer, decreased expression of granzyme B, perforin, and IFN-γ in infiltrating T cells, NK cells, and NKT cells can be detected [36]. To regulate the expression of perforin, granzyme B, CD95, CD95 ligand, and TRAIL, IFN-γ may increase the mRNA expression levels of these factors to facilitate cell death in the targeted cells [4, 15, 67, 78, 80]. Additionally, IFN-γ potentiates CD95- and TRAIL-induced apoptosis by enhancing downstream caspase-8 protein expression [43]. IFN-γ plays an essential role in the induction of cytolytic activity in CTLs, most likely by affecting the membrane expression of the interleukin (IL)-2 receptor [29]. Autocrine IFN-γ stimulation on CD4^+^ T cells promotes adaptive immune response by augmenting cell survival and cytokine secretion during T cell activation [61]. IFN-γ is also secreted from human invariant NKT cells as well as γδ T cells to promote tumor-associated antigen-specific CTL responses [37, 54, 69]. For CTL responses, the induction of MHC class I and transporter associated with antigen processing (TAP) can be elicited by IFN-γ stimulation in target cancer cells. Additionally, in activated CTLs, IFN-γ is effectively produced to promote differentiation and activation [64]. As a Th1 cytokine, IFN-γ has been shown to trigger M1-polarized differentiation of anticancer macrophage phenotype but not tumor-promoting M2 macrophages with immunosuppressive properties [20]. Thus, IFN-γ plays a key immunomodulatory role in cancer immunosurveillance.

### Cancer immune escape

Cancer cells that survive immunosurveillance by using so-called immune evasion are crucial for carcinogenesis. After immunoediting, cancer cells harbor a variety of strategies to escape immunosurveillance. Basically, the processes of intrinsically inducing tolerance in cancer cells themselves and extrinsically inducing resistance to cytotoxic immune effector cells are initially elicited during tumorigenesis [6, 19, 79]. Under immune escape, an active process that stimulates suppressive factors to cause inhibitory and/or cytotoxic effects on CTLs, NK cells, NKT cells, and γδ T cells have been extensively documented both directly and indirectly.

The intrinsic pathways to escape immunosurveillance in cancer cells by changing their immunogenicity are largely used during tumorigenesis. Downregulating tumor antigen presentation by causing decreased levels of MHC class I, TAPs, tapasin, and the proteasome subunits of latent membrane proteins is common in most cancers [28, 30, 79]; however, the mechanisms involved require further investigation. Two of these mechanisms are abnormal genetic and epigenetic regulation and unresponsiveness to IFNs, both of which are required for inducing expression of proteins related to tumor antigen presentation processes [28]. Activation of oncogenic processes such as increased activity of the oncogenes c-Myc and Bcl-2 and decreased activity of the tumor suppressor genes p53 and PTEN may strengthen the survival responses of cells resisting cell death, and the immunological cytotoxicities from tumor-infiltrating immune suppressive cells are speculated to be decreased and/or stopped [33]. Therefore, a combinatorial therapy targeting oncogenic signaling pathways in cancer cells can concomitantly enhance the CTL response [83].

Several extrinsic ways of inducing the production of humoral factors by tumors and preventing the infiltration of numerous suppressor cells against effector cytotoxic cells are stimulated in the progression of resistance due to tumorigenesis [3, 7, 21, 22, 50, 79, 82]. By secreting and/or expressing immunosuppressive factors such as IL-10, transforming growth factor (TGF)-β, prostaglandin E2 (PGE_2_), indoleamine-pyrrole 2,3-dioxygenase (IDO), galectins, and programmed death ligand 1 (PD-L1), cancer cells can locally block CTL-mediated cytotoxicity by causing antigen/MHC loss and T cell dysfunction [88, 90]. IL-10 is primarily produced by monocytes, M2 cells, Th2 cells, mast cells, Tregs, MDSCs, and MSCs in response to inflammation, autoimmunity, infection, and tumorigenesis [40, 55, 62, 75]. Some cancer cells express more IL-10, which is correlated with cancer progression from the radial to vertical growth phase as well as with the development of metastatic competence [38]. Stimulation of IL-10 activates STAT3-mediated suppressors of cytokine signaling (SOCS) three expression, which confers anti-inflammatory responses by inhibiting Th1 cell proliferation and altering Th1/Th2 differentiation [23, 93].

PD-L1 (also known as CD274 or B7 homolog 1 (B7-H1)) is abundantly expressed in various human cancers [17] and can activate PD-1 signaling to induce T cell exhaustion in a tumor microenvironment [58, 87]. The blockade of tumor-associated PD-L1, which can increase inactivation and/or apoptosis of T cells and result in immune evasion of cancers [18], is currently used as a powerful immunotherapy that acts in a manner similar to targeting immune checkpoints such as PD-1 and cytotoxic T-lymphocyte-associated protein (CTLA) 4 [58, 73, 87].

Tumor microenvironment contains the infiltration of tumor-promoting immunosuppressive cells such as TAMs, CD4^+^CD25^+^FOXP3^+^ Tregs, and MDSCs, which are necessary for tumorigenesis [7, 82]. M2 TAMs, which display pro-tumoral functions, including the expression of various growth factors, promotion of angiogenesis, and suppression of CTL responses, are commonly present in malignant tumors, which are linked to a poor prognosis in those patients with breast cancer, ovarian cancer, some types of glioma, and lymphoma [5]. M2 TAMs are therefore targeted for anticancer therapies [25]. The differentiation of Tregs by IL-10, IL-35, and TGF-β, all of which are regulated by the expression and activation of the transcription factor Foxp3, is crucial for tumorigenesis [14]. Regarding Treg-secreted IL-10 and TGF-β-suppressed T cell responses, depleting Tregs or inhibiting their immune inhibitory actions can enhance anticancer effects [14]. MDSCs abolish the immune responses during tumor progression [42]. These cells can inhibit efficient anticancer T cell responses by inducing Treg activity and M2 differentiation (via IL-10 and TGF-β), depriving amino acid metabolism in T cells (via arginase 1), releasing cytotoxic oxidizing molecules to deactivate local T cells (via hydrogen peroxide and peroxynitrite), interfering with T cell migration (CC chemokine ligand (CCL) two inactivation and disintegrin and metalloproteinase domain-containing protein 17-mediated CD62L cleavage), causing T cell death (via galectin-9), and inhibiting NK activation (through TGF-β) [16, 26, 42]. Targeting MDSCs is currently implemented as a cancer immunotherapy.

### Cancer cells show hyporesponsiveness to IFN-γ-dependent immunosurveillance

As described above, cancer cells develop immune evasion strategies to extrinsically escape from IFN-γ-dependent immunosurveillance by releasing immunosuppressive factors as well as recruiting immunosuppressive cells; however, it has been speculated that the generation of cellular tolerance in cancer cells against IFN-γ-mediated anticancer signaling occurs during tumorigenesis. Basically, IFN-γ effectively increases MHC class I and cytotoxic proteins related to CTL responses to strengthening the anticancer activity. However, the loss of MHC class I and cytotoxic priming proteins may result from cellular hyporesponsiveness to IFN-γ [28]. According to this hypothesis, changes in the activation of IFN-γ signaling should be considered as an alternative escape pathway from IFN-γ-dependent immunosurveillance in tumorigenesis.

For controlling IFN-γ signaling, three types of proteins act as the negative regulator to inhibit IFN-γ: Src homology 2-containing phosphatase (SHP) 2, the protein inhibitors of activated STATs, and SOCSs [89]. SOCS1 and SOCS3, which are positively induced by IFN-γ-activated JAK-STAT pathway, may in turn affect JAK activity and STAT recruitment to turn off signaling after ligand binding [91]. IL-10 is able to induce SOCS3 expression to block IFN-γ signaling by competing with the binding of JAK to IFNRGs [39]. Alternatively, the protein tyrosine phosphatase SHP2 dephosphorylates JAKs and IFNGR1 to halt the signaling of IFN-γ. Additionally, STAT1 activation can be directly downregulated by SHP2 [92]. SHP2 phosphorylation at Tyr542 and Tyr580 on its carboxyl-terminus indicates an activated status of SHP2 in response to growth factor receptor activation. Nevertheless, the post-translational modifications of SHP2 remain unclear. Regarding the negative regulatory effects of SOCSs and SHP2, it has been speculated that tumors with SHP2 hyperactivation and SOCS overexpression may gain the potential escape mechanisms from IFN-γ-initiated immune defense responses.

For the development of leukemia, breast cancer, oral cancer, laryngeal cancer, lung cancer, liver cancer, and gastric cancer, the aberrant expression and activation of SHP2 has been identified as oncogenic for facilitating cancer cell hyper-proliferation through a mechanism involving activation of MAPK/extracellular signal-regulated kinase (ERK) signaling [8, 9, 94]. However, the mechanisms and mutations involved in SHP2 activation remain unclear. Epidermal growth factor stimulates increased SHP2 protein tyrosine phosphatase activity to mediate paxillin dephosphorylation, ERK activation, and cell migration [13]. Furthermore, the SHP2 inhibitor SPI-112Me enhances IFN-γ signaling and subsequent related pathways including STAT1 activation, IFN-sensitive response element transactivation, p21 expression, and cell growth inhibition. Hyporesponsiveness to IFN-γ is speculated to be accompanied by SHP2 activation.

*Helicobacter pylori* (*H. pylori*) is the first identified carcinogenic bacterium that is a well-known inducer of gastric tumorigenesis. Upon *H. pylori* infection of gastric epithelial cells, the bacteria-secreted virulent factor CagA binds with SHP2 to cause its direct activation, and SHP2 is oncogenic for the transformation of gastric epithelial cells [35]. Our recent studies have demonstrated that CagA-regulated SHP2 activation facilitates IFN-γ hyporesponsiveness in gastric epithelial cells during *H. pylori* infection [85]. For gastric tumorigenesis, SHP2 activation not only induces MAPK/ERK-mediated cell transformation but also promotes IFN-γ hyporesponsiveness as an escape from IFN-γ-dependent cancer immunosurveillance. Interestingly, both human AGS gastric epithelial adenocarcinoma cells and PC14PE6/AS2 lung epithelial adenocarcinoma cells show IFN-γ hyporesponsiveness [12, 48, 76, 77, 81]. There were no differences in the expression of IFNGR1 and IFNGR2. In response to IFN-γ-induced STAT1 phosphorylation at Tyr701, IRF1 transactivation, an increase in STAT1/IRF1 protein levels, CD54 expression, inducible nitric oxide (NO) synthase (iNOS)/NO induction, cell growth inhibition, and cytotoxicity, both AGS and PC14PE6/AS2 cells are extremely resistant to the hyperactivation of SHP2. Genetically and pharmacologically inhibiting SHP2 can reverse IFN-γ signaling and cellular regulation as described above. These results confirm the negative role of SHP2 in reducing IFN-γ signaling and illustrate a possible immune evasive role of SHP2 against IFN-γ-dependent cancer immunosurveillance. SHP2 may act as an intracellular factor that induces tolerance and/or senescence in cancer cells in response to IFN-γ stimulation.

For SHP2 regulation in response to IFN-γ treatment, we have previously demonstrated that either glycogen synthase kinase (GSK)-3β activation [74] or autophagic induction [10, 11] facilitates IFN-γ signal transduction by inhibiting SHP2. In contrast, aberrant oncogenic PI3K activation as well as a decrease in the expression of the tumor suppressor PTEN induces AKT activation accompanied by GSK-3β inactivation and SHP2 activation. Therefore, PI3K/PTEN/AKT/GSK-3β/SHP2-facilitated IFN-γ resistance can be identified in cancer cells [12, 76, 77]. Regarding the notion that oncogenic galectin-3 may prompt cellular transformation through the activity of Ras and PI3K/AKT [56, 71], we found that galectin-3 is overexpressed in AGS cells and is involved in modulating AKT phosphorylation at Thr308 independent of activation of either PI3K or 3-phosphoinositide-dependent protein kinase-1 [76]. Manipulating galectin-3 expression can alter the signaling of AKT/GSK-3β/SHP2 to affect the cellular hyporesponsiveness to IFN-γ. According to these findings, oncogenic signaling pathways related to AKT-mediated GSK-3β inactivation are speculated to be important for SHP2 activation as well as IFN-γ hyporesponsiveness. Targeting the possible oncogenic signals in malignancies not only reduces cell proliferation and cell survival but also modulates the cellular tolerance of escape from IFN-γ-dependent cancer immunosurveillance. As reported by previous studies on a defective response of MHC I expression in IFN-γ-resistant AGS cells [1, 2], future studies are needed to determine whether targeting SHP2 can reverse the lack of immunogenicity in cancer cells and whether the IFN-γ-induced MHC class I and cytotoxic factors related to the CTL responses can be overturned.

### Implications in cancer immunotherapy

Re-activating tumor-suppressing cells, including NK, NKT, γδ T cells, and CTLs, is crucial for therapeutic effects of immune checkpoint blockade. Targeting CTLA4 and PD-1 on CTLs and its principal ligand PD-L1 on cancer cells is currently used in clinical trials [58, 59]. However, several issues need to be considered for determining a successful cancer checkpoint immunotherapy [52]. For the status of T cell activation, biomarkers are needed to evaluate the efficacy of immune checkpoint inhibition [52]. For the susceptibility of cancer targeting, cancer cells may also show immune escape from re-activated CTL responses following immunotherapy. As we know, immune checkpoint blocking overcomes T cell exhaustion and improves CTL responses, including IFN-γ production and IFN-γ-dependent cancer immunosurveillance [58]. Regarding the role of IFN-γ signaling in cancer cells in the setting of immune checkpoint remains unknown, a recent study demonstrated that tumors with genomic defects or decreases in IFN-γ signaling show cellular hyporesponsiveness to immune checkpoint inhibition [27]. Therefore, signaling of IFN-γ may be a prognostic target as well as a biomarker for a successful immunotherapy in patients with treatment of immune checkpoint inhibitors. Furthermore, the therapeutic combination approaches concurrently by using chemotherapy, angiogenic blockers, immune-checkpoint inhibitors, immunostimulatory agents, and cancer vaccines, which have effectively worked on IFN-γ signaling, are speculated to be achieved in the near future [59].

## Conclusions

**Fig. 1:**
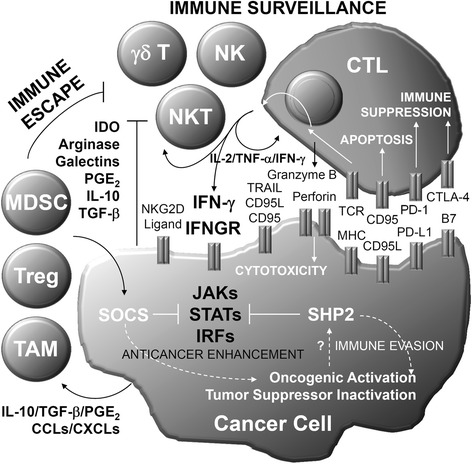
Immune surveillance and escape from IFN-γ-dependent anticancer activity. CCL: CC chemokine ligands; CXCL: CXC chemokine ligands; CTL: cytotoxic T lymphocytes; CTLA: cytotoxic T-lymphocyte-associated protein; IDO: indoleamine-pyrrole 2,3-dioxygenase; IFN: interferon; IFNGR: IFN-γ receptors; IL: interleukin; IRF: IFN-regulating factors; JAK: Janus kinase; MDSC: myeloid-derived suppressor cell; MHC: major histocompatibility complex; NK: natural killer; PD-L1: programmed death ligand 1; PGE: prostaglandin E; SHP: src homology-2 containing phosphatase; SOCS: suppressors of cytokine signaling; STAT: signal transducer and activator of transcription; TAM: tumor-associated macrophage; TAP: transporter associated with antigen processing; TGF: transforming growth factor; TNF: tumor necrosis factor; TRAIL: TNF-related apoptosis-inducing ligand; Treg: regulatory T cell

As summarized in Fig. 1, after MHC/TCR- and NKG2DL-mediated pathways, immune surveillance driven by anticancer immune cells (such as NK, NKT, CTL, and γδT cells) and immune factors (such as IL-2, TNF-α, IFN-γ, granzyme B, perforin, TRAIL, and CD95L) confers anti-tumorigenesis. In addition to perforin/granzyme B- and TRAIL/CD95L-mediated cytotoxicity, IFN-γ/IFN-γ receptor signaling causes the Jak/STAT/IRF1-mediated pathway to induce anticancer enhancement effects by upregulating MHC molecules as well as cytotoxic factors. Therefore, avoiding immune destruction, so called immune escape from anticancer immune cells and immune factors, is important for tumor evasion as one of the hallmarks of cancer. For immune escape, the extrinsic pathways, by recruiting and activating immunosuppressive tumor-associated TAMs, Tregs, and MDSCs via IL-10/TGF-β/PGE_2_/CCLs/CXC chemokine ligands-regulated mechanisms, are important to defeat immune surveillance. Immunosuppressive factors such as IDO, arginase, galectins, PGE_2_, IL-10, and TGF-β are able to inactivate anticancer immune cells. Additionally, the intrinsic pathways for immune escape can be initiated by immune checkpoint co-inhibitory signals such as B7/CTLA4- and PD-L1/PD-1-mediated immune suppression as well as by the induction of apoptosis in CTLs through a CD95L/CD95-mediated pathway.

By blocking immune checkpoint signaling and depleting immunosuppressive cells, targeting immune evasion is now a potent strategy against tumorigenesis. IFN-γ-facilitated elimination is a key process of elimination in immunosurveillance; however, there are diverse mechanisms available to cancer cells to escape from IFN-γ-dependent anticancer signaling. Soluble factors in the microenvironment and immunosuppressive cells are speculated to attenuate the IFN-γ response of NK, NKT, CTL, and γδT cells; it is hypothesized that oncogenic signals, such as SOCSs and SHP2, in malignancies also cause cellular hyporesponsiveness, such as immune evasion, in response to IFN-γ anticancer activities, including cancer cell growth inhibition, cytotoxicity, and MHC class I expression. Although exogenous IFN-γ treatment confers limited results in clinical therapy due to its side effects on systemic inflammation, concurrently reversing production of IFN-γ in tumor-suppressing cells and signaling of IFN-γ in cancer cells can be utilized to evaluate the therapeutic efficacy after anticancer treatment, particularly in immune checkpoint-based therapy.

## Abbreviations

ATM: Ataxia telangiectasia mutated
ATR: ATM and Rad3-related
CD: Cluster of differentiation
CTL: Cytotoxic T lymphocytes
CTLA: Cytotoxic T-lymphocyte-associated protein
ER: Endoplasmic reticulum
ERK: Extracellular signal-regulated kinases
ETosis: Extracellular trap cell death
FOXP3: Forkhead box P3
GAS: IFN-γ-activated site
GSK-3β: Glycogen synthase kinase-3β
IDO: Indoleamine-pyrrole 2,3-dioxygenase
IFN: Interferon
IFNGR: IFN-γ receptors
IL: Interleukin
iNOS: Inducible nitric oxide synthase
IRF: IFN-regulating factors
JAK: Janus kinase
MAPK: Mitogen activated protein kinase
MDSC: Myeloid-derived suppressor cell
MHC: Major histocompatibility complex
NK: Natural killer
PAD: Protein arginine deiminase
PD-L1: Programmed death ligand 1
PGE2: Prostaglandin E2
PTEN: Phosphatase and tensin homolog deleted on chromosome
ROS: Reactive oxygen species
SHP: Src homology-2 containing phosphatase
SOCS: Suppressors of cytokine signaling
STAT: Signal transducer and activator of transcription
TAM: Tumor-associated macrophage
TAP: Transporter associated with antigen processing
TGF: Transforming growth factor
Th1: Type 1 T helper cell
TNF: Tumor necrosis factor
TRAIL: TNF-related apoptosis-inducing ligand
Treg: Regulatory T cell
